# L’Enseignement Et L’Organization De L’Art Dentaire Aux E’tats Unis

**Published:** 1889-10

**Authors:** 


**L'Enseignement et L'Organization de L'Art Dentaire aux E'tats Unis.** Rapport Addressé a Monsieur le Ministre de L'Instruction Publique par le Dr. Kuhn. Pp. 299. Paris: Octave Doin, Editor. 8 Place de L'Odeon. 1888.

This report to the Minister of Public Instruction, on Dentistry in the United States, is based on observations made by Dr. Kuhn during his visit to this country at the time of the last International Medical Congress.

It contains a review of the dental laws of the several States and Territories, and the courses of study of the different dental colleges. Noteworthy among the latter is the report on the colored dental school connected with the Meharry Medical Department of Central Tennessee College, which is the only school of the kind in the world.

The report closes with a high appreciation of the position to which dentistry has reached in this country, and hopes that, little by little, they may some day approach us. "This hope should not be considered chimerical." The report is a masterpiece of thorough work.

